# Paraneoplastic hypercalcemia in clear cell ovarian adenocarcinoma

**DOI:** 10.3332/ecancer.2012.271

**Published:** 2012-10-02

**Authors:** S Lewin, D Dezube, AK Guddati, K Mittal, F Muggia, Paula Klein

**Affiliations:** 1 Department of Pediatrics, New York Presbyterian/Weill Cornell Medical Center, New York, NY, USA; 2 Brown Alpert School of Medicine, Brown University, Providence, RI, USA; 3 Department of Internal Medicine, St. Vincent’s Medical Center, Bridgeport, CT, USA; 4 Division of Gynecological and Obstetric Pathology, Department of Pathology, New York University School of Medicine, New York, NY, USA; 5 Division of Medical Oncology, Department of Medicine, New York University School of Medicine, 160 East 34th Street, New York, NY 10016, USA; 6 Beth Israel Cancer Center, Albert Einstein College of Medicine, New York, NY, USA

**Keywords:** *hypercalcemia*, *clear cell adenocarcinoma*, *paraneoplastic*, *ovarian*, *PTHrP*

## Abstract

**Background:**

Hypercalcemia has been reported in association with a number of malignancies, but it is an unusual manifestation of ovarian cancer. This finding at presentation (possibly aggravated by oral calcium intake) led to discovery of a clear cell carcinoma of the ovary. The implications and pathophysiology of this association are reviewed.

**Case report:**

Following presentation with abdominal symptoms, this premenopausal woman was found to have bilateral adnexal masses and hypercalcemia. Her parathormone-related polypeptide was found to be elevated. After surgery and staging, she received adjuvant carboplatin and paclitaxel (later substituted by docetaxel). She has done well on her long-term follow-up.

**Conclusions:**

This rare paraneoplastic manifestation of ovarian cancer may be associated with long-term survival if discovered at an early stage. In this instance, further benefit may have been obtained from adjuvant platinum-based chemotherapy.

## Introduction

Hypercalcemia is one of the most frequently encountered paraneoplastic syndromes, occurring in 20–30% of patients with cancer [1]. Historically, hypercalcemia has been associated mainly with squamous cell carcinoma of the bronchus, carcinoma of the breast and multiple myeloma [2]. Calcium metabolism is a major extent under the control of parathyroid hormone (PTH), which acts to modulate calcium reabsorption in the distal tubule and on its mobilization from bone. Uncontrolled production of a similar substance to parathormone by the tumour was postulated to be a potential cause for hypercalcemia of malignancy by Albright in 1941 in a patient diagnosed with renal cell carcinoma with metastasis to the bones presenting with hypercalcemia concurrently with hyperphosphatemia [3]. He noted that the hypercalcemia appeared to be in excess of what would be expected from the presence of bone metastases. This led to further exploration of ‘pseudohyperparathyroidism’ and eventually resulted in the discovery of PTH-related protein (PTHrP), a polypeptide hormone isolated from tumours associated with hypercalcemia that exhibits considerable homology with PTH [4]. Similar to PTH, PTHrP exerts its effects by inducing increased bone resorption and reducing renal excretion of calcium [5]. PTHrP is physiologically secreted during pregnancy by the amnion, chorion and placenta, and has also been shown to be cyclically expressed during normal menstrual cycle [6, 7]. PTHrP production in malignancy has been associated with activating mutations in the Ras pathway and with altered expression of viral or cellular transcription factors [8]. Although Albright’s original description was in association with clear cell carcinoma of the kidney, the tumour types most commonly associated with humoral hypercalcemia of malignancy are lung (26–28%), breast (24–26%) and multiple myeloma (5–8%) [2, 9]. Here, we discuss a patient presenting with humoral hypercalcemia that subsequently led to identification of an early stage clear cell ovarian adenocarcinoma resulting in an apparently curative outcome.

## Case presentation

The patient is a 44-year old school teacher who presented with gastrointestinal disturbances including nausea, intermittent vomiting, abdominal pain, constipation and anorexia. Gynecological history included onset of menarche at age 11, first pregnancy at age 21 and biannual vaginal ultrasound surveillance for a cyst on the right ovary and a fibroid on the left ovary; menorrhagia had been present for approximately two years prior to her current symptoms. Her ‘functional’ bleeding was managed by Orthotricyclen (ethinyl estradiol and norgestimate) for three months, followed by Provera (medroxyprogesterone) (10 mg qd) for one cycle. Her mother had been diagnosed with ductal carcinoma in situ (DCIS) and was receiving tamoxifen; there was no family history of malignancy or endocrine diseases. Perhaps because of participation in her mother’s care, the patient became aware of issues concerning osteopenia and had initiated on her own preventive measures such as calcium supplements to improve her ‘bone health’.

Notably, after the onset of symptoms, the vaginal ultrasound revealed the new finding of a 9-cm solid adnexal mass on the right side with a 3-mm fluid-filled cyst. A serum calcium level of 11.7 mg/dl (normal: 8.0–10.0 mg/dl) with a rise to 12.5 mg/dl three weeks later suggested a hypercalcemia of malignancy. On this second, blood sample serum PTH was <5.0 pg/ml (normal: 65 pg/ml), and a concurrent urine analysis demonstrated the urinary calcium to be 470 mg/24 h (normal: <250 mg/24 h), further pointing to such a diagnosis as opposed to primary hyperparathyroidism, milk-alkali syndrome or hypervitaminosis D. A serum protein electrophoresis was obtained, only notable for an elevated alpha-1-globulin of 0.47 g/dl (indicative of a reactive process or inflammation); the vitamin D1,25-dehydrogenase was 110 pg/ml (almost twice the normal upper limit). Urine protein electrophoresis was negative for Bence-Jones proteins and monoclonal antibodies ruling out multiple myeloma. Further, determination of parathormone-related polypeptide was found to be elevated (laboratory value no longer available) and confirmatory of a paraneoplastic manifestation. At referral to the medical oncology clinic, the patient’s calcium level had risen to 13 mg/dl, and the intact PTH was <1.00 pg/ml; these were all recorded while the patient’s albumin levels were consistently within normal limits (4.3 g/dl). This sample was also remarkable for elevated tumour markers, with a CA 15-3 of 494.9 U/ml and a CA 125 of 159.7 U/ml, consistent with an epithelial malignancy; another epithelial marker of malignancy, CA 19-9, was within normal limits. The patient was advised to cease her over-the counter 2,000 mg daily calcium supplements (taken for the past 10 years). By now, physical exam revealed a palpable, mobile mass in the right lower quadrant. On computerized tomography of the chest, abdomen and pelvis, definite findings were confined to a large, heterogeneous pelvic mass and an enlarged uterus, potentially contiguous with the mass. Attention was also drawn to small non-calcified and non-specific sub-centimeter nodules in the lung, liver and spleen. The patient received a bisphosphonate (pamidronate 90 mg IV) over 2 h to control the hypercalcemia and was referred to a surgeon specializing in gynecological oncology.

**Figure 1:**
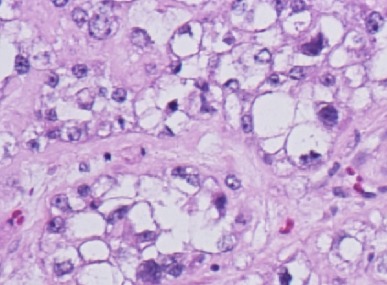
Clear cell adenocarcinoma of the ovary, showing cells with clear cytoplasm and pleomorphic high-grade nuclei (Hematoxylin & Eosin, ×200).

Six weeks after her initial presentation, the patient underwent a total abdominal hysterectomy with bilateral salpingo-oophorectomy. Her pre-operative blood work, done approximately two weeks after cessation of calcium supplements, demonstrated a normal serum calcium level of 10.1 mg/dl. Intraoperative findings included a 12-cm right ovary that was described as mostly solid, with a cystic component and associated necrosis. The right fallopian tube was adherent to the mass. The tumour had both solid and cystic components. Microscopic examination showed clear cell adenocarcinoma. The tumour cells had clear cytoplasm and pleomorphic high-grade nuclei (Figure 1). No transcapsular spread was noted, but positive cytology in pelvic washings led to staging as 1C. No tumour was found in the fallopian tubes, left ovary, multiple peiritoneal biopsies, omentum, right pelvic nodes and pre-caval lymph nodes. Following an uneventful recovery, the patient’s tumour markers dropped markedly: 12 days post-surgery, the patient’s CA 125 had dropped to 95.1 U/ml and her CA 15-3 to 117.6 U/ml. The patient received six cycles of paclitaxel (175 mg/m2) and carboplatin AUC 6 every 3 weeks. Due to development of grade 2 neuropathy, paclitaxel was replaced by docetaxel (75 mg/m2) until the completion of her regimen. Two months after surgery and thereafter, the serum calcium CA 125 and CA 15-3 have been within normal limits and she continues in follow-up care doing well.

## Discussion

Ovarian carcinomas account for 6% of all malignancies in women and most often present at an advanced stage. The clear cell subtype of ovarian surface epithelial-stromal tumours is over-represented among stage I epithelial tumours of the ovary and while known to be more aggressive than the serous, endometrioid and mucinous subtypes, a five-year survival rate exceeding 65% (and as high as 81.7% in a 2012 series) may be expected in stage IC patients that underwent full staging. With spread beyond the ovary, survival of five years is exceptional with a prognosis distinctly poorer as compared to the other subtypes of epithelial ovarian carcinomas. Moreover, resistance to conventional platinum-based therapy has often been emphasized. Fortunately, our patient was diagnosed at stage 1C, partly because her paraneoplastic hypercalcemia became symptomatic (possibly aggravated by her calcium intake) and she underwent potentially curative surgery early and additionally treated with the taxane-platinum-based chemotherapy reported to contribute to the management of early stage clear-cell histologies [10, 11].

Gynecological malignancies have been shown to be associated with humoral hypercalcemia of malignancy with clear cell of the ovary histology being most prominent [12]. Ovarian cancer has been associated with several paraneoplastic syndromes, including the recently described thrombocytosis [13], cerebellar degeneration [14] and encephalitis [15]. Clear cell adenocarcinomas are characterized by cells that appear clear on H&E staining due to increased glycogen content in the cytoplasm. The nuclei are usually high grade, and there is usually a high-mitotic count, attesting to the relatively aggressive nature of this malignancy. The risk factors for ovarian carcinoma include nulliparity, family history and heritable mutations. Because of the patient’s mother carrying a diagnosis of in-situ breast cancer, genetic testing was done and she was found to be negative for BRCA mutations. Women between the ages of 40–59 years who had taken oral contraceptives, as did our patient, actually have a decreased risk of developing ovarian cancer. Hypercalcemia is a hallmark of a rare ovarian tumour designated ‘Small cell carcinoma of hypercalcemic type’ [16, 17]. These tumours typically occur in adolescents and young women. Microscopic features of small cell carcinoma were not seen in the current case.

Hypercalcemia is a condition that has potentially serious sequelae, including alterations in mental status, renal dysfunction and electrocardiogram abnormalities such as prolongation of the PR interval and shortening of the QT interval. Severe hypercalcemia can result in coma. Prognosis of hypercalcemia associated with malignancy is poor; the 1-year survival rate is 10–30%. This case demonstrates that successful management of the underlying malignancy leads to resolution of its paraneoplastic manifestations. It also illustrates the importance of a thorough evaluation and vigilance of hypercalcemia, and the inclusion of ovarian carcinoma in the differential diagnosis.
